# Molecular Dynamics Investigations of Human DNA-Topoisomerase I Interacting with Novel Dewar Valence Photo-Adducts: Insights into Inhibitory Activity

**DOI:** 10.3390/ijms25010234

**Published:** 2023-12-23

**Authors:** Jessica Di Martino, Manuel Arcieri, Francesco Madeddu, Michele Pieroni, Giovanni Carotenuto, Paolo Bottoni, Lorenzo Botta, Tiziana Castrignanò, Sofia Gabellone, Raffaele Saladino

**Affiliations:** 1 Department of Ecological and Biological Sciences, Tuscia University, Largo dell’Università snc, 01100 Viterbo, Italy; jessica.dimartino@unitus.it (J.D.M.); saladino@unitus.it (R.S.); 2 Department of Health Technology, Technical University of Denmark, 2800 Kongens Lyngby, Denmark; s230158@dtu.dk; 3 Department of Computer Science, “Sapienza” University of Rome, P.le Aldo Moro, 5, 00185 Rome, Italypieroni.1704202@studenti.uniroma1.it (M.P.); bottoni@di.uniroma1.it (P.B.); 4 Preclinic and Osteoncology Unit, Biosciences Laboratory, IRCCS Istituto Romagnolo per lo Studio dei Tumori (IRST) “Dino Amadori”, 47014 Meldola, Italy

**Keywords:** human topoisomerase I, molecular dynamics, Dewar valence photo-adducts, ligand–receptor interaction

## Abstract

Chronic exposure to ultraviolet (UV) radiation is known to induce the formation of DNA photo-adducts, including cyclobutane pyrimidine dimers (CPDs) and Dewar valence derivatives (DVs). While CPDs usually occur at higher frequency than DVs, recent studies have shown that the latter display superior selectivity and significant stability in interaction with the human DNA/topoisomerase 1 complex (TOP1). With the aim to deeply investigate the mechanism of interaction of DVs with TOP1, we report here four all-atom molecular dynamic simulations spanning one microsecond. These simulations are focused on the stability and conformational changes of two DNA/TOP1-DV complexes in solution, the data being compared with the biomimetic thymine dimer counterparts. Results from root-mean-square deviation (RMSD) and root-mean-square fluctuation (RMSF) analyses unequivocally confirmed increased stability of the DNA/TOP1-DV complexes throughout the simulation duration. Detailed interaction analyses, uncovering the presence of salt bridges, hydrogen bonds, water-mediated interactions, and hydrophobic interactions, as well as pinpointing the non-covalent interactions within the complexes, enabled the identification of specific TOP1 residues involved in the interactions over time and suggested a potential TOP1 inhibition mechanism in action.

## 1. Introduction

Continuous exposure to ultraviolet (UV) radiation constitutes a well-known risk factor associated with multiple detrimental effects, including skin ageing, epigenetic alterations, compromised immune system, angiogenesis, nucleotide base mutations, and the risk of developing melanoma [1]. The effect of UV radiation on pyrimidine DNA nucleobases, particularly thymine, leads to the formation of excited energy states, thereby generating cyclobutane pyrimidine dimers (CPDs), pyrimidine-(6-4)-pyrimidone (6-4 PP), and Dewar valence (DV) photo-adducts [2]. The most prevalent photo-adducts are CPDs, which occur three to four times more frequently [3].

UV-A radiation is directly adsorbed by DNA, triggering the generation of reactive radicals that oxidize nucleobases and activate damage pathways involving electron transfer processes associated with pheomelanin [4,5]. To prevent DNA mutations, cells activate a complex network of repair mechanisms, including the recognition of photo-radiation-induced damage [6], cell cycle arrest [7], excision of photo-adducts, and apoptosis [8,9]. The tumorigenesis, including the formation of melanoma, can be triggered by the failure of defence mechanisms in preserving genetic integrity [10].

Photo-adducts also interact with topoisomerase 1 (hTop1p) [11], resulting in the activation of the topoisomerase cleavage complex, which inhibits re-ligation processes [12,13]. Specifically, hTop1p becomes trapped by oligonucleotide sequences containing photo-adducts, resulting in the formation of single-strand breaks (SSBs) [14].

Melanoma represents one of the most aggressive forms of skin cancer, yet therapeutic options remain limited. With recent surge in incidence, the quest for specific treatments has become of vital importance [15]. The use of nanotechnology in the development of targeted therapies against melanoma offers significant advantages and could open new horizons in clinical therapy [16,17,18]. As an example, lignin nanoparticles (LNPs) have been investigated as drug delivery systems due to their favourable biocompatibility, biodegradability, multifunctionality, and UV protective capabilities [19].

In this context, the objective of current research is to develop an integrated approach that exploits the regulated release of photo-adducts from nanoparticles.

In a recent study [19], we reported that CPD, (6-4) PP, and DV thymine photo-adduct analogues characterised by the presence of a carbon spacer between two nucleobase units were effective inhibitors of two melanoma cancer cell lines, namely SK-MEL28 and RPMI7951 [19]. The presence of an aliphatic spacer between the two modified nucleobases increased the stability of novel photo-adduct analogues with respect to the natural phospho-diester counterpart. In addition, the possibility to control the administration of photo-adduct analogues encapsulated inside lignin nanoparticles was reported [19], highlighting a new possibility for drug delivery. Preliminary in silico molecular docking showed that the interaction of DVs **8a** and **8b** with the hTop1p/DNA duplex (TOP1) was more efficient than the parent thymine dimers **4a** and **4b**, demonstrating greater anti-melanoma activity [18]. In particular, DVs **8a** and **8b** showed inhibitory activity higher than known anticancer drugs. Once encapsulated in LNPs, these compounds maintain their activity, offering gradual release and protection against photodegradation.

On the basis of these data, we report here a set of simulations and computational analyses in order to deeply investigate the interaction of DVs **8a** and **8b** with TOP1, analysing the types of interactions and evaluating their duration and stability.

All the main analyses of this article were performed with software tools optimized for high-performance computing (HPC) to handle large volumes of data in bioinformatics [20]. HPC bioinformatics infrastructures enable the examination of complex biological macromolecules [21,22], as well as the analysis of vast quantities of novel biological data [23,24].

For our analyses, we used ligand–receptor complexes formed by hTop1p in association with DNA. During the docking phase, we incorporated the modified dimers (**4a**, **4b**, **8a**, and **8b**), replacing the original camptothecin portion in the reference structure (PDB ID: 1T8I). Table 1 provides an overview of the structure of the photo-adducts, their SMILES strings, and the binding affinity for each one of the best poses. Subsequently, we conducted molecular dynamics (MD) simulations in order to study the dynamic behaviour of molecular systems over time, considering all the entities involved (compound, protein, and water) as flexible entities [25,26,27,28]. The MD simulation had a duration of 1 µs for the solvated ternary complexes, enabling us to assess the stability of DVs **8a** and **8b** with respect to the parent thymine dimers (**4a** and **4b**). We have provided comprehensive details of the MD protocol, including energy minimization, thermalization, and molecular dynamics, in the dedicated methodology section. As a result, we obtained trajectories of the solvated complexes in saline solution. These data were used for subsequent phases of analysis, focused on studying the interactions between the compounds and TOP1 over time. In addition, the MD-ligand-receptor HPC pipeline [22] was utilised to profile the protein–compound interactions. This allowed us to quantify the duration of each interaction over time, followed by their classification (e.g., hydrogen bonds, hydrophobic interactions), and identification on the basis of atom tuples. Finally, we applied principal component analysis (PCA) to identify important collective variables that influence compound–receptor dynamics [29,30]. Furthermore, integrating free-energy calculations with PCA has proven to be a valuable approach, offering a comprehensive view of the conformational landscape accessible to a protein. This integrated analysis improves our understanding of compound–receptor dynamics, providing detailed insights into system behaviour.

## 2. Results and Discussion

### 2.1. Classical Molecular Dynamics Simulation

**Figure 1:**
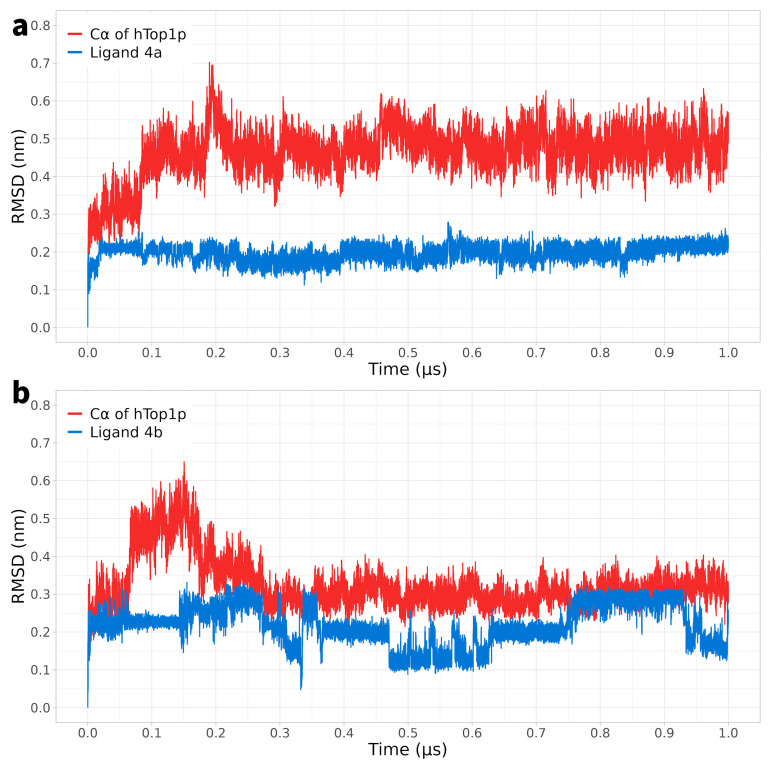
Panel (**a**): MD simulation of the solvated complex htop1p-**4a**, the root mean square deviation (RMSD) of the protein’s Cα atoms is depicted in red, while the RMSD of the ligand’s atoms **4a** is shown in blue. Panel (**b**): MD simulation of the solvated complex htop1p-**4b**; the root mean square deviation (RMSD) of the protein’s Cα atoms is depicted in red, while the RMSD of the ligand’s atoms **4b** is shown in blue.

For a period of 1 μs, classical MD simulations were conducted by analysing the 1T8I receptor of hTop1p (without CPT) complexed with compounds **4a**, **4b**, **8a**, and **8b** to investigate the overall stability of each system. It is important to note that the initial DNA used did not contain modified thymine dimers. During the docking phase, the modified dimers were added, replacing the camptothecin portion. Regarding compound **4a**, it was observed that the root-mean-square deviation (RMSD) of the Cα atoms of the protein started at approximately 0.4 nm and was stabilised with an average value around 0.6 nm after approximately 0.1 microseconds. Meanwhile, the RMSD of the compound remained constant and close to 0.2 nm almost immediately (Figure 1a).

In the case of compound **4b**, the RMSD of the Cα atoms of the protein started at a higher value of approximately 0.6 nm, which was stabilised after about 0.2 microseconds to a value of 0.4 nm. Meanwhile, ligand **4b** showed a slightly lower average RMSD of 0.3 nm than **4a** (Figure 1b).

**Figure 2:**
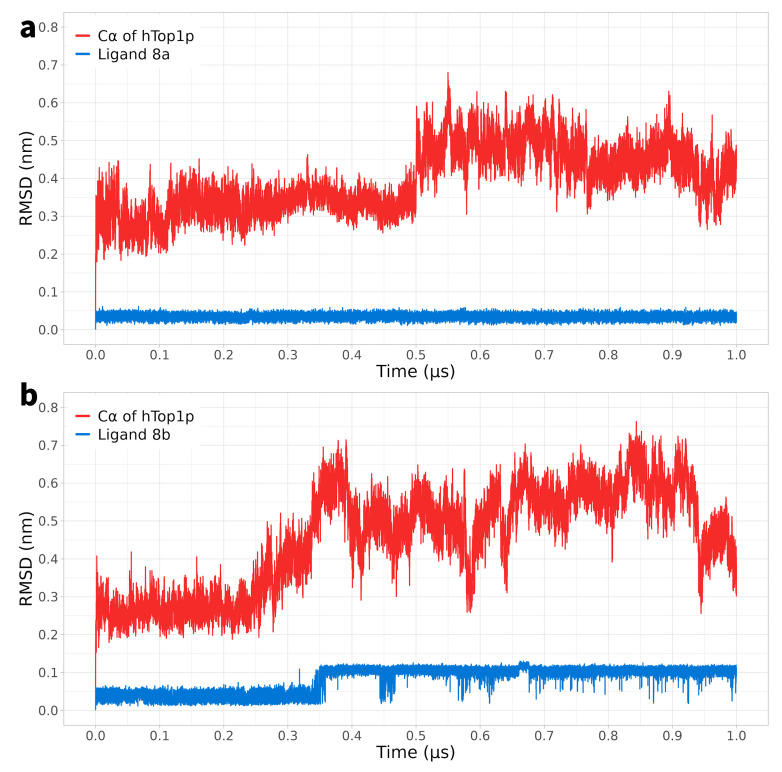
Panel (**a**): MD simulation of the solvated complex htop1p-**8a**; the root mean square deviation (RMSD) of the protein’s Cα atoms is depicted in red, while the RMSD of the ligand’s atoms **8a** is shown in blue. Panel (**b**): MD simulation of the solvated complex htop1p-**8b**; the root mean square deviation (RMSD) of the protein’s Cα atoms is depicted in red, while the RMSD of the ligand’s atoms **8b** is shown in blue.

In the case of compounds **8a** and **8b**, the RMSD of the Cα atoms of the protein showed an initial fluctuation but reached values of 0.6 nm at different times (after 0.5 microseconds for **8a** and after 0.4 microseconds for **8b**) (Figure 2a). It is important to note that, unlike compounds **4a**-**b**, DVs **8a** and **8b** remained consistently close to 0 nm and only experienced small variations after a certain period of time (0.4 microseconds for **8b**) (Figure 2b).

Thus, while in the case of compounds **4a** and **4b**, the RMSD of the protein’s Cα atoms displayed significant fluctuations during the simulation, suggesting greater variability in the conformation of the protein–ligand complex over time, DVs **8a** and **8b** exhibited a tendency to stabilise after a brief initial fluctuation, and the RMSD remained constant throughout the simulation. These data suggest greater stability and less conformational variability in the complexes of DVs **8a** and **8b** compared to that of compounds **4a** and **4b**.

We focused on the analysis of the flexibility of amino acid residues (root-mean-square fluctuation, RMSF) within the hTop1p domain, as these residues constitute the main components of the binding site for the compounds. Elevated RMSF values indicate significant flexibility of the residues during the course of the simulation.

**Figure 3:**
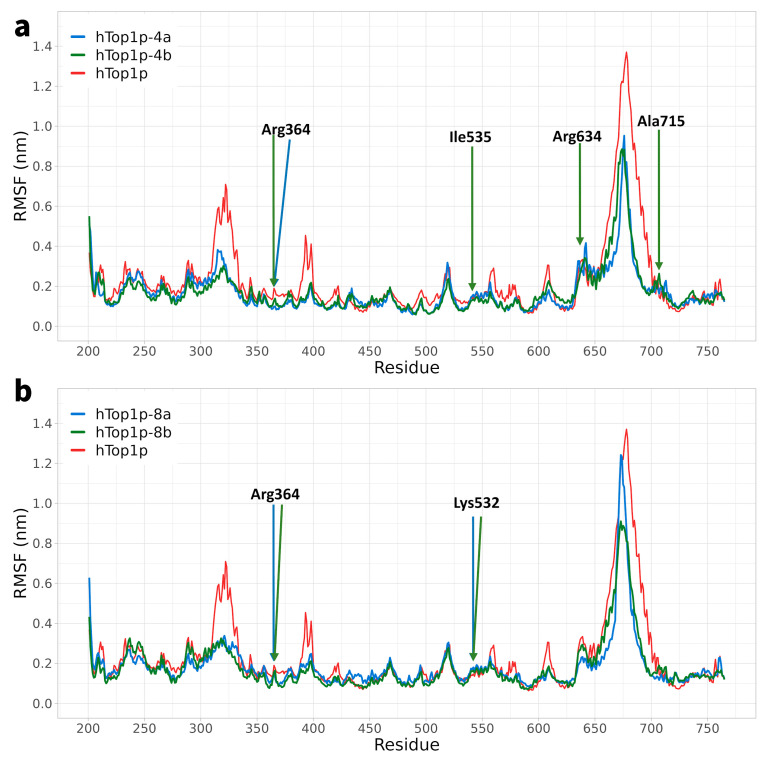
RMSF (root-mean-square fluctuation) of the core domain of hTop1p. Panel (**a**), blue line: RMSF of the core domain of htop1p in complex with compound **4a**. In green: RMSF of the core domain of htop1p in complex with compound **4b**. In red: RMSF core domain of htop1p. Panel (**b**), blue line: RMSF of the core domain of htop1p in complex with compound **8a**. In green: RMSF of the core domain of htop1p in complex with compound **8b**. In red: RMSF core domain of htop1p.

**Figure 4:**
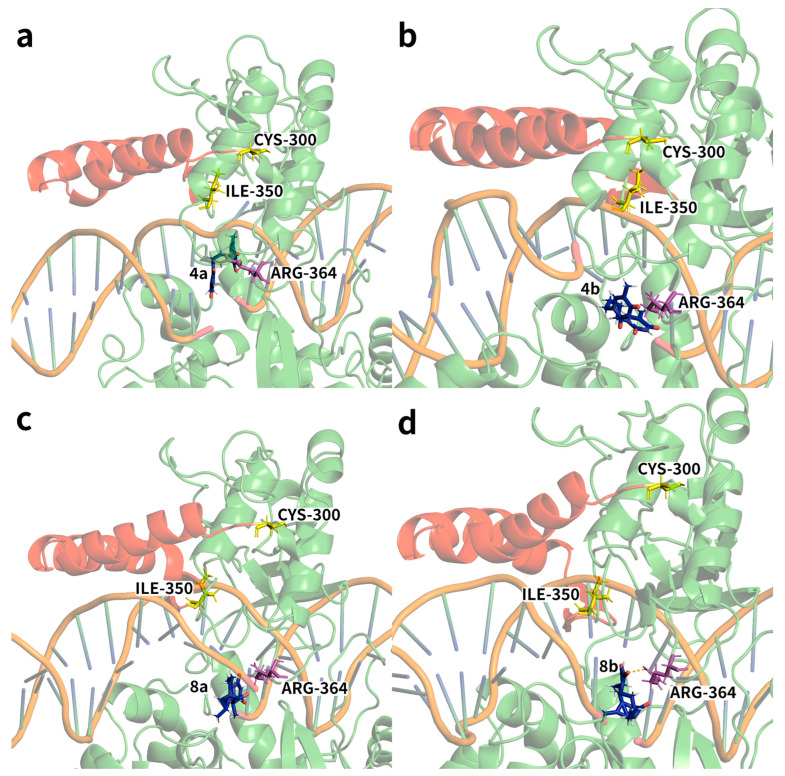
Four snapshots are provided from the simulations with the four ligands, (**a**) **4a**, (**b**) **4b**, (**c**) **8a** and (**d**) **8b** to highlight the range of residues 300–350. The ligands are shown in green, but in particular residues 301–349 are highlighted in red, while residues 300 and 350, respectively, are highlighted in yellow to mark the beginning and end of the interval under study. In this interval, a significant reduction in flexibility is observed from the RMSF plots. The ligand and the first interacting residue (chromophore Arg364) near the region under study are displayed using a ‘ball-and-stick’ model.

In the hTop1p without compounds, a slight increase in RMSF values was observed in the region between amino acids 300–350 and 380–400, suggesting increased mechanical destabilisation in these regions (Figure 3). However, these fluctuations of hTop1p were mitigated in the presence of compounds. Particularly in the region between residues 300 and 350, we observed a significant reduction in flexibility for ligands **4b**, **8a**, and **8b**, while for ligand **4a**, the decrease in flexibility was less marked. In this context, we provide snapshot depictions (four panels of Figure 4) that identify the region 300–350 (highlighted in red for residues 351–349; residues 300 and 350, respectively, highlighted in yellow to mark the beginning and end of the region) in the four MD simulations with the corresponding ligand highlighted, and the first residue proximal to the region and interacting with the ligand (the chromophore Arg364). Furthermore, it is important to highlight that no significant differences emerged when comparing the RMSF values in the central domain of hTop1p when the protein was bonded to compounds. In the RMSF graphs reporting the interactions between protein and compounds **4a** and **4b** (Figure 3a), no significant fluctuations were observed. It is evident that the lines representing the protein alone and the compound bound to the protein fluctuated synchronously, suggesting greater stability in the receptor–ligand systems. The amino acid range 630–700 exhibited fluctuations, as it represents the linker domain of our protein, known for its free fluctuations and extensive movements [31,32]. This domain does not constitute contact points with our compounds. After this interval, the protein re-established contact with the compounds, and the fluctuations returned to stability. Comparison of the RMSF variations between compounds **4a** and **4b** and DV **8a** and **8b** (Figure 3) highlighted a subtle difference in flexibility in specific regions, particularly in the 650 region. This divergence is attributable to the lack of stable bonds in the surrounding area, in particular with Arg634 and Ala715, present in compounds **4a** and **4b**. Their absence in complexes **8a** and **8b** helped to give that region a slight increase in flexibility. For the remainder of the RMSF of DVs **8a** and **8b** (Figure 3b), the RMSF trends were similar to those observed in compounds **4a** and **4b**.

### 2.2. Ligand Receptor Binding Interactions as a Function of Time

By running the MD-ligand-receptor pipeline, we identified ligand–receptor interactions that were present for most of the simulation time. These interactions were categorised by bond type and by the duration of the linkage throughout the entire simulation. These analyses revealed a significant difference in the stability of interactions between compounds **4a** and **4b** compared to DVs **8a** and **8b**.

**Figure 5:**
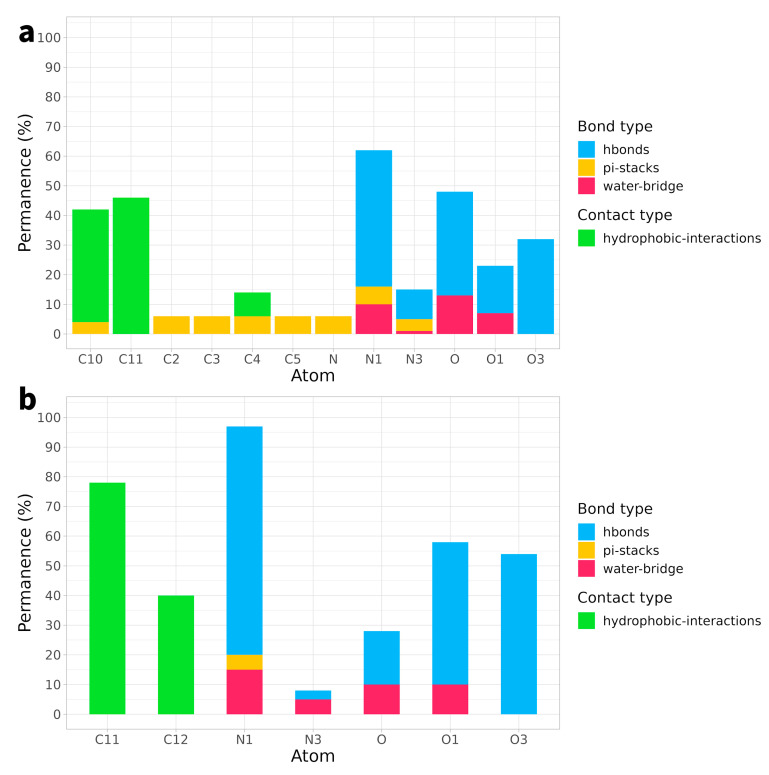
Types of bond and interactions established by each individual atom of compound **4a** (Panel (**a**)) and **4b** (Panel (**b**)) as a function of simulation time. In the bar graph, the atoms involved in the interactions are represented on the *x*-axis, the time in timeframes of the simulation is shown on the *y*-axis, and the colour indicates the different types of bonds or interactions. The graph is based on a 5% threshold for atom residence.

Interactions between a ligand and its receptor play a crucial role in the effectiveness of a compound as a therapeutic agent. For compounds **4a** and **4b**, various types of bonds or interactions., including hydrogen bonds, hydrophobic interactions, pi-stack bonds, and hydrogen bridges, were observed. However, it is important to note that, despite the variety of bond types, most of them had a relatively short interaction time. In particular, hydrogen bonds, crucial for strong interactions, were maintained for only 28% of the timeframes in the case of **4a** and approximately 40% for **4b** (Figure 5). For the rest of the bonds, the duration was even shorter. The brief duration of these bonds can impact the overall stability of interactions.

**Figure 6:**
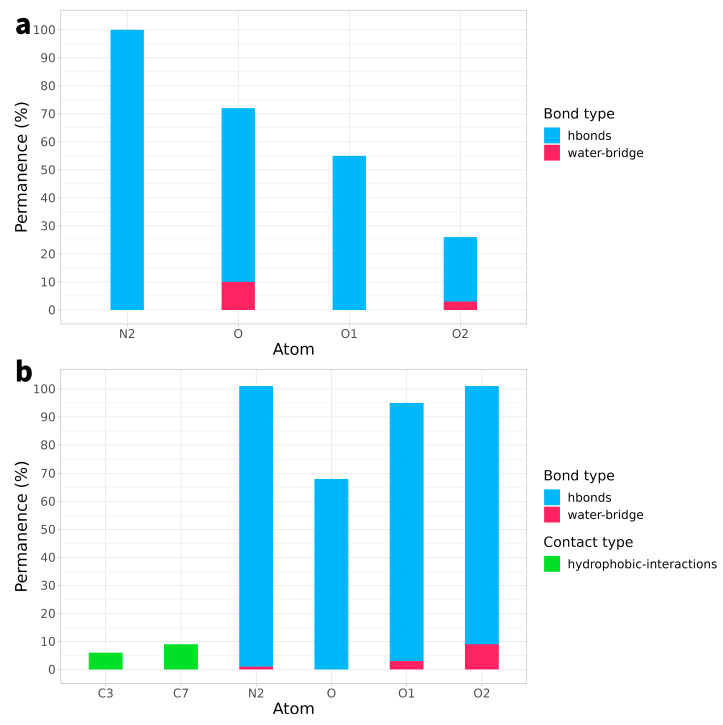
Types of bond and interactions established by each individual atom of compound **8a** (Panel (**a**)) and **8b** (Panel (**b**)) as a function of simulation time. In the bar graph, the atoms involved in the interactions are represented on the *x*-axis, the time in timeframes of the simulation is shown on the *y*-axis, and the colour indicates the different types of bonds or interactions. The graph is based on a 5% threshold for atom residence.

On the other hand, in the case of compounds **8a** and **8b**, hydrogen bonds still represent the predominant type of interaction with the receptor, but what significantly stands out is their remarkable duration. In the case of **8a**, these bonds were maintained on average for over 63% of the simulation time, with very high peaks, such as in the case of residue N2, where they were maintained for 99% of the time. Similarly, compound **8b** exhibited hydrogen bonds that persisted for approximately 89% of the simulation time, with timeframes nearing 100 in several residues, such as N2, O1, and O2 (Figure 6).

These results appear to align with the previous RMSD findings, highlighting a clear difference in the stability of interactions between compounds **4a**–**b** and compounds **8a**–**b**. While the interactions in compounds **4a**–**b** were short-lived, compounds **8a**–**b** showed stable interactions for a significantly higher percentage of the simulation time, indicating greater interaction stability.

Next, we conducted a detailed investigation of the non-covalent interaction between amino acid residues and nucleobases in the 1T8I receptor, with specific attention to Arg364. In the case of compound **4a**, the primary interactions involved Arg364 and Lys751, along with nucleobases DC111, DC112, and DT10. Table 2 shows the percentages of permanence of the most relevant non-covalent interactions between the atoms of compound **4a** and the amino acids or nucleotides of the 1T8I receptor. The asterisks specifically indicate hydrophobic interactions, highlighted to provide further distinction between the different types of bonds analysed. While some interactions, such as N1-DC112 and C10/C11-DT10, proved to be relatively persistent (interaction times of 24%, 42%, and 39%, respectively), the overall stability of these interactions remained moderate. In the case of compound **4b**, the predominant interactions involved atoms O and N1, which were in contact with Arg364 for 23% and 42% of the total simulation time, respectively. Additionally, N1 established a significant interaction with DC112 for 62% of the time. Residue C11 primarily interacted with amino acid Ile535, persisting for 77% of the simulation time (Table 3). In the case of compound **8a**, the interactions included prevalently the O atoms with the Arg364 for 50% of the simulation time, O1 atoms forming bonds with Lys532 for 54% of the time, and N2, interacting with DT10 for 87% of the simulation time (Table 4). Compound **8b** exhibited remarkably stable interactions, with O primarily interacting with Lys532 for 55% of the time, and O1 forming enduring bonds for a remarkable 91% of the time with residue DC112, which simultaneously interacted with ligand residue N2 for over 97% of the simulation time (Table 5).

**Figure 7:**
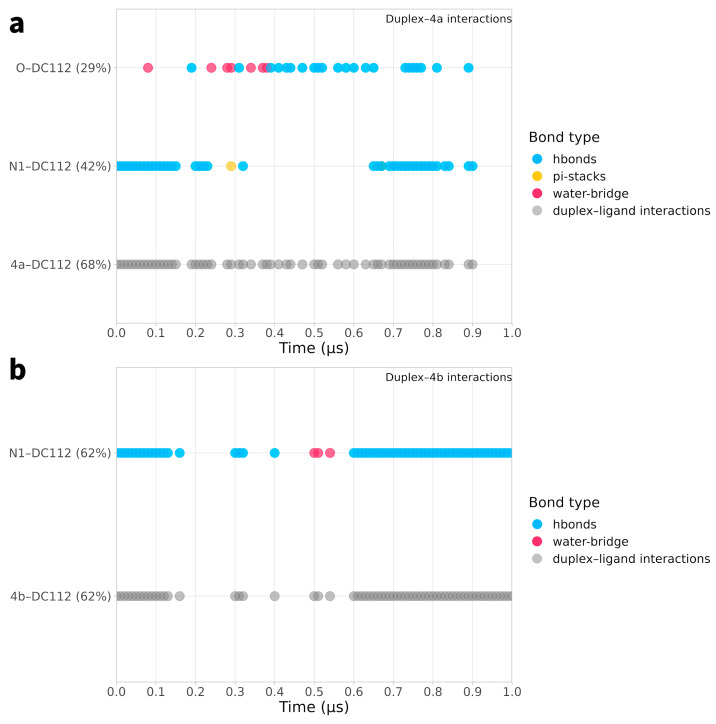
Time-dependent interactions of key nucleotides (the most interacting nucleotides with the four ligands as described in Table 2, Table 3, Table 4 and Table 5) with each of the four studied ligands. Panels (**a**,**b**) correspond to the key residues interacting with ligands **4a** and **4b**, respectively.

**Figure 8:**
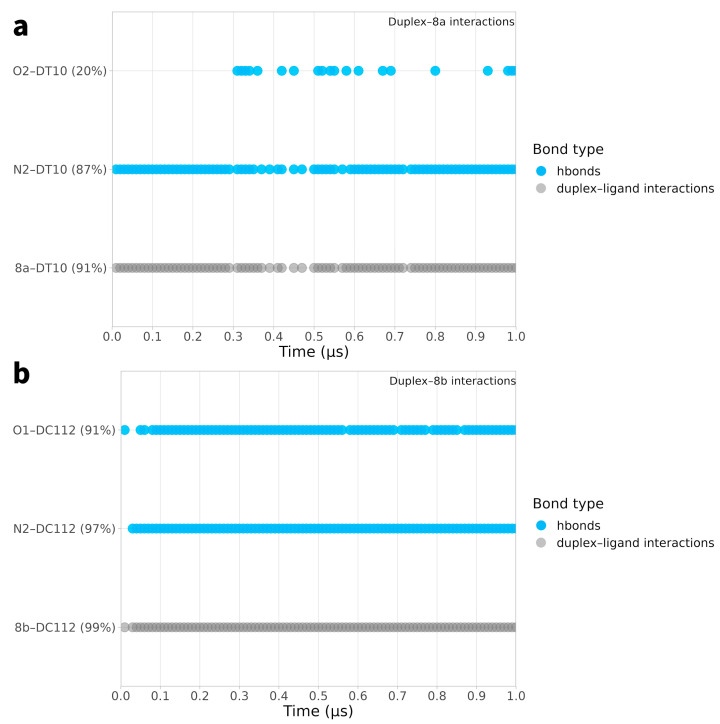
Time-dependent interactions of key nucleotides (the most interacting nucleotides with the four ligands as described in Table 2, Table 3, Table 4 and Table 5) with each of the four studied ligands. Panels (**a**,**b**) correspond to the key residues interacting with ligands **8a** and **8b**, respectively.

In Figure 7 and Figure 8, the temporal interactions of key nucleotides (the most interacting nucleotides are described in Table 2, Table 3, Table 4 and Table 5) with each of the four studied ligands are depicted. As can be observed, nucleotide DC112 plays a crucial role in the stability of the duplex–ligand interaction, as demonstrated in the MD simulations with ligands **4a**, **4b**, and **8b**. Particularly in the plot of the interaction with ligand **8b**, the substantial contribution of the duplex–**8b** interaction is evident, which, along with the ligand’s interactions with the protein, imparts a particularly pronounced inhibitory role. Equally important for the stability of ligand **8a** is the interaction with nucleotide DT10, which is maintained for a significant portion of the simulation.

**Figure 9:**
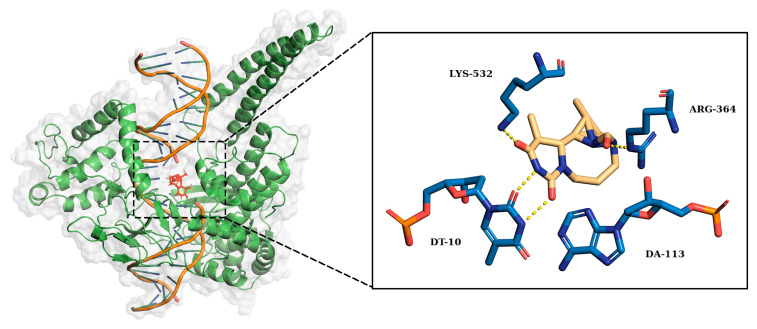
Right: 3D structures of compound **8a** lodged in the receptor pocket. Left: the residues most involved in forming interactions with the amino acids and nucleotides of the 1T8I receptor are highlighted.

**Figure 10:**
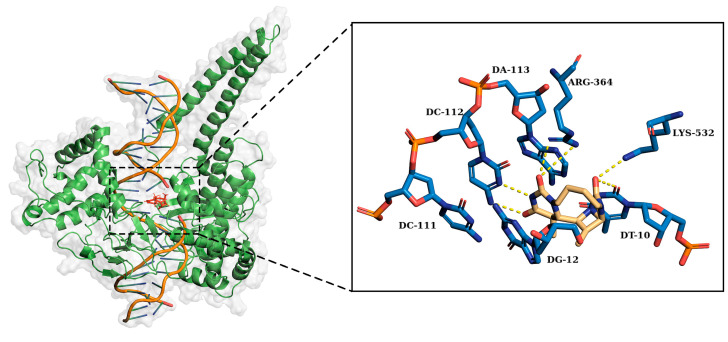
Right: 3D structures of compound **8b** lodged in the receptor pocket. Left: the residues most involved in forming interactions with the amino acids and nucleotides of the 1T8I receptor are highlighted.

Figure 9 and Figure 10 show the chemical structure of compounds **8a** and **8b** and highlight the main residues responsible for direct binding to the receptor.

### 2.3. PCA and Free-energy Landscape Analysis

The conformation of proteins plays an essential role in defining their function and the required rigidity, particularly in the ligand-binding region. A crucial indicator of the ligand’s inhibitory capability is the persistent stability of the catalytic site throughout the entire dynamic simulation. This stability can be assessed through principal component analysis (PCA), allowing for a detailed examination of protein movements during their interaction with the ligand throughout the simulation.

The PCA was performed on the trajectories of the Cα atoms of the htop1p protein. Subsequently, the covariance matrix was derived from these trajectories, and the motion profiles of the pivotal residues obtained from the dynamic simulations were projected onto the first two principal components (PCs). This approach provides precision in analysing the macro-molecular motions of the enzyme itself, as well as the specific amino acid residues.

**Figure 11:**
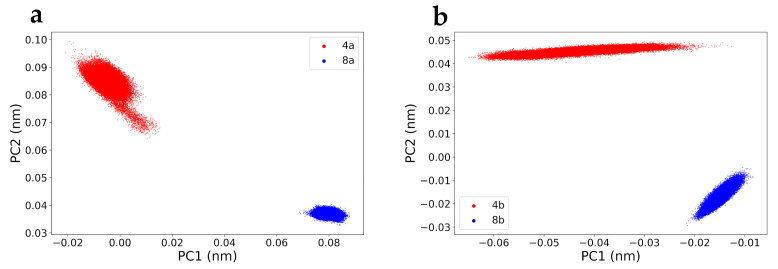
PCA constructed for protein–ligand as a function of the best projections of the MD trajectory onto the first (PC1) and second (PC2) eigenvectors, respectively. Panel (**a**): compound **4a** (red) and **8a** (blue). Panel (**b**): compound **4b** (red) and compound **8b** (blue).

From the analysis of molecular interactions using the MD-ligand-receptor pipeline, key residues that play a fundamental role in the ligand interaction can emerge. The overall flexibility of the complexes was measured along PC1 and PC2. The projection of PC1 and PC2 is depicted in Figure 11 for all complexes. As reported in Figure 11, complexes involving DVs **8a** and **8b** exhibited less overall movement compared to the others, while complexes involving **4a** and **4b** covered a wider conformational space. This result is consistent with previously performed analyses, like the four RMSDs. Therefore, complexes involving DVs **8a** and **8b** showed kinetically relevant states and were confined to a smaller area than their parent counterparts, indicating a less flexible structure. The greater movement of complexes involving **4a** and **4b** also led to the confinement of a broad region in conformational space. Consequently, the overall PCA analysis reveals that compounds **8a** and **8b** exhibited significantly strong interactions.

Free-energy landscape analysis was performed to evaluate the conformation distribution and structure stability of the protein–ligand complex of the most significant trajectories from the dynamic simulation. The conformational models were studied via free-energy landscape (FEL) with respect to PC1 and PC2. The stability of the complex is measured on the basis of the relative energy minima, where the lower free energy of the complex represents the greater conformational stability of the complex. The lower energy basin is indicated with a more intense blue colour. Free-energy contour maps for all four protein–ligand systems were created here. The Gibbs FEL was obtained using the principal components as reaction coordinates and is shown in Figure 11. The different accessible conformational states of proteins with compounds **4a**, **4b**, **8a**, and **8b** during the simulation were mapped onto the landscape of free energy together with PC1 and PC2.

**Figure 12:**
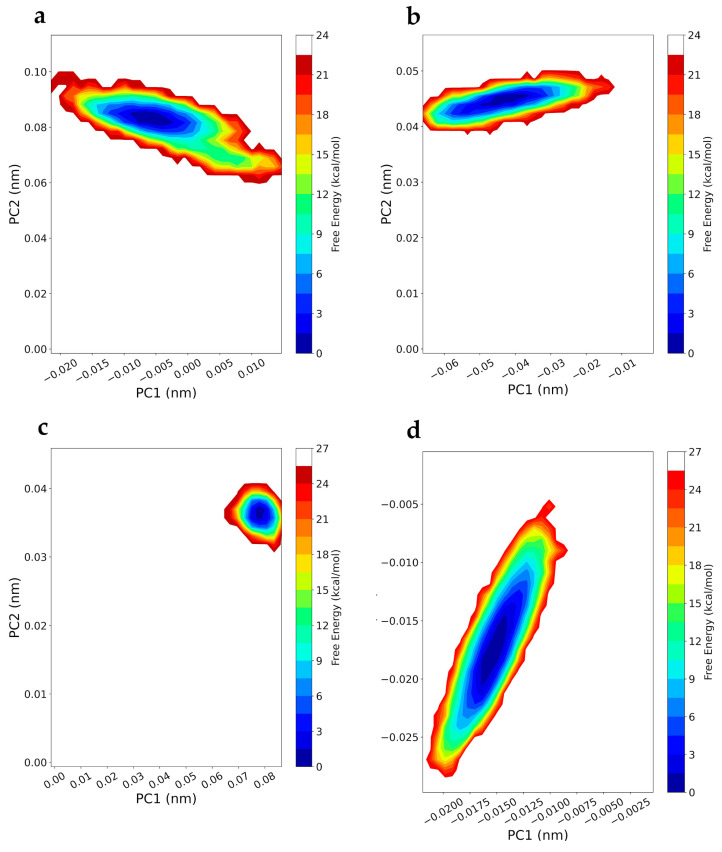
Free-energy landscape of the ligand-docked complex of compounds **4a** (panel (**a**)), **4b** (panel (**b**)), **8a** (panel (**c**)), and **8b** (panel (**d**)) as a function of MD trajectory projections onto the first (PC1) and second (PC2) eigenvectors, respectively. The colour bar represents the relative free energy value in kcal mol.

As highlighted in Figure 12, complexes involving DVs **8a** and **8b** exhibited a well-defined global minimum energy basin, corresponding to their conformational state, and were distributed more compactly compared to the ensemble of other cases (as highlighted by FEL data). Conversely, complexes involving compounds **4a** and **4b** displayed significant conformational changes and a broader, more dispersed, basin as observed in the FEL plots. This clearly indicates destabilisation of the complex. The increased flexibility leads to higher fluctuations and weaker binding with the ligand. These data strongly suggest that compounds **8a** and **8b** interact very efficiently with the ligands, occupying more stable conformations.

## 3. Materials and Methods

### 3.1. Data Preparation (In Silico Molecular Docking)

The receptor used in our study was based on the crystal structure obtained from X-ray diffraction of human DNA topoisomerase I in complex with camptothecin (CPT) and covalently linked to a 22-base pair DNA duplex (PDB ID: 1T8I) [33]. This structure served as the foundation for our docking investigations. To initiate the docking studies, we eliminated ligands that were originally located within the active site of the 1T8I model. The 3D spatial coordinates for compounds **8a**, **8b**, **4a**, and **4b**, represented in SMILES format (as shown in Table 1), were generated using Open Babel software, version 2.3.2 [34]. The visual representations presented in our research were created using PyMol software v. 2.5.7 available from http://pymol.sourceforge.net (retrieved on 10 October 2021) (Table 6). Our in silico molecular docking simulations aimed to predict the formation of molecular complexes, specifically the binding of htop1p with ligands **8a**, **8b**, **4a**, and **4b**, respectively. To validate our docking methodology, we first re-docked the crystallised pose of CPT into the receptor. This validated protocol was then applied to dock all the compounds. The preparation of both the receptor and compounds for docking was performed using AutoDockTools v. 1.5.6 [35], and the results were saved in pdbqt format. For receptor preparation, polar hydrogens were added, and Kollman charges were employed to calculate the partial charges. Meanwhile, for the compounds, the Gasteiger charges method was used to assign the partial charges.

The docking analyses were carried out using AutoDock Vina software [36,37], producing 20 potential binding poses for each compound. The selection of the most suitable docked conformations was based on binding affinity and spatial alignment. To define the search space for docking, a grid box measuring 20 × 20 × 20 units was oriented around the CPT binding site.

### 3.2. Classical Molecular Dynamics Simulation

In our study, we conducted classical molecular dynamics (MD) simulations using Gromacs version 2021.2 (available at https://gromacs.org/2021.2/.html accessed on 18 October 2023) and employed the AMBER99 force field [38]. Compound topologies were generated with Acpype software v. 2023.10.27 [39], which incorporates the AMBER atomic definitions. The initial structures for MD simulations were based on the 1T8I-ligand complexes obtained from our molecular docking experiments. These systems were centred within a simulation box and hydrated using the TIP3P water model [40]. Ions were added to neutralise the system. We initiated energy minimization using the steepest descent method, setting a limit of 50,000 steps, and terminating when forces decreased to below 1000 kJ/mol/nm. This was followed by an NVT equilibration phase lasting 1 ns at a constant temperature of 300 K, employing the V-rescale algorithm. Subsequently, we conducted an NPT equilibration phase of 2 ns, utilising the Berendsen barostat to maintain pressure at 1 bar. The particle mesh Ewald method was applied to handle long-range electrostatic interactions within the system. The four prepared complexes (htop1p in complex with **8a**, **8b**, **4a**, and **4b**, respectively) were then subjected to a 1 µs production MD simulation, using a timestep of 2 fs and saving trajectories every 10 ps.

### 3.3. Analysis of Ligand–Receptor Interactions

Ligand–receptor binding interactions were assessed using the MD-ligand-receptor pipeline [22], a specialised software designed to investigate ligand–receptor interactions using molecular dynamics trajectories. The program interfaces with GROMACS to extract conformational data from the trajectories and employs PLIP [41] to analyse ligand–receptor binding interactions. MDLR is equipped with parallel processing capabilities, optimising resource utilisation on a high-performance computing cluster and generating the tables and plots presented in the preceding sections of this article.

### 3.4. PCA Analysis and Free-energy Calculation

The principal component analysis (PCA) was carried out using the tools provided by GROMACS. Briefly, the covariance matrix of the atomic positions is built from the MD simulations on a selected group of atoms (usually Cα). From the diagonalization of such a matrix, a set of eigenvectors and associated eigenvalues is obtained. The eigenvectors represent the principal motion directions of the system and, therefore, they are used to describe the “essential” protein modes, which often represent the functional ones. The covariance matrices, calculated using the GROMACS covar tool, were constructed from the Cα atoms of proteins of the MD trajectories. The GROMACS anaeig tool was used to calculate the 2D projections with respect to the first two eigenvectors and eigenvector components to the selected eigenvectors. The GROMACS sham tool was used to calculate the free energy landscape ΔG along the first two principal components, $V_{1}$ and $V_{2}$, in units of $k_{B}T$.

## 4. Conclusions

The results of our research provide an in-depth understanding of the interaction between Dewar valence isomers (DVs) and the DNA/topoisomerase 1 (hTop1p) complex, with significant implications for the development of targeted antitumour therapies.

Initially, we conducted 1 microsecond molecular dynamics simulations to study the stability of DV complexes compared to thymine dimers. The DV complexes (**8a** and **8b**) exhibited significantly greater stability compared to their thymine-dimer-based counterparts (**4a** and **4b**). This stability was evidenced by low values of the root-mean-square deviation (RMSD) of the Cα atoms of the protein and ligand in the DV complexes. This remarkable stability can be attributed to a range of non-covalent interactions, hydrogen bonds, hydrophobic interactions, and water-mediated bonds [36]. These results confirm the presence of stronger interactions within the DV complexes, implying a higher degree of interaction stability compared to the thymine-dimer-based counterparts.

The analysis of the interactions was conducted using the MD-ligand-receptor pipeline, revealing a clear disparity in the stability of interactions between DV complexes and thymidine-dimer-based complexes. In DV complexes, interactions were observed to be more durable, with a particular emphasis on hydrogen bonds. These findings align with RMSD analyses, suggesting greater interaction stability within the DV complexes.

Subsequent analyses showed the involvement of specific receptor residues, with particular attention to Arg364, already shown to be a key residue for the inhibitory action of the chromophore CPT. These residues emerged as key players in the interactions between DV compounds and the receptor, forming stable bonds.

Furthermore, through the application of principal component analysis (PCA) simulations and free-energy landscape (FEL) analysis, we further elucidated that DV complexes displayed a more compact distribution of conformations within a clearly defined global minimum energy basin, whereas thymidine-dimer-based complexes exhibited higher conformational variability.

In summary, DV complexes stand out for their stability, compact conformations, and robust interactions. These results underscore the revolutionary potential of DV-like compounds as therapeutic agents. Their stability and effectiveness within DNA/hTop1p complexes suggest new avenues for the treatment of skin tumours, such as melanoma.

This study represents a significant contribution to our understanding of molecular interactions in therapeutic contexts and holds promise for substantial impacts on the development of innovative and targeted antitumour therapies.

## Figures and Tables

**Table 1:** Compounds of interest: 2D chemical structure, SMILES format, and binding affinity during docking. ^a^ Docking analysis (AutoDock Vina software, AutoDock Vina 1).

Compound Name	SMILES Strings	2D Structure	Binding Affinity (Kcal/mol) ^a^
**4a**	C1(C(=CN(C(N1)=O)CCCN2C=C(C(NC2=O)=O)C)C)=O	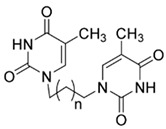 n = 1	−7.1
**4b**	C(CCCN1C=C(C(NC1=O)=O)C)N2C(NC(C(=C2)C)=O)=O	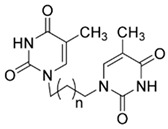 n = 2	−7.3
**8a**	O=C(N1)N2CCCN3C(N4C3=O)C(C)=C4C2C(C)(O)C1=O	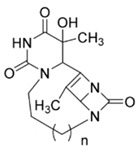 n = 1	−7.9
**8b**	O=C(N1)N2CCCCN3C(N4C3=O)C(C)=C4C2C(C)(O)C1=O	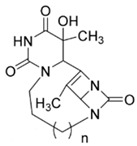 n = 2	−8.8

**Table 2:** Permanence in percentage of the most relevant non-covalent interactions, with asterisks (*) indicating hydrophobic interactions between the atoms of compound **4a** and the amino acids or nucleotides of the 1T8I receptor.

	Arg364	Thr718	Leu721	Lys751	DC111	DC112	DG12	DT10
O					18%	29%		
O1	13%						14%	
O3		8%		24%				
N					5%			
N1	9%				12%	42%	8%	
N3							5%	
C2					5%			
C3					5%			
C4					5% + 8% *			
C5					5%			
C10								4% + 35% *
C11			10% *					39% *

**Table 3:** Permanence in percentage of the most relevant non-covalent interactions, with asterisks (*) indicating hydrophobic interactions between the atoms of compound **4b** and the amino acids or nucleotides of the 1T8I receptor.

	Arg364	Ile535	Arg634	Ala715	DC112	DG12
O	23%					
O1	11%					51%
O3						50%
N						5%
N1	42%				62%	9%
N3						5%
C2						5%
C3						5%
C4						5%
C5						5%
C11		77% *				
C12			13% *	25% *		

**Table 4:** Permanence in percentage of the most relevant non-covalent interactions between the atoms of compound **8a** and the amino acids or nucleotides of the 1T8I receptor.

	Arg364	Lys532	DA113	DT10
O	50%		17%	
O1		54%		
O2				20%
N2		19%		87%

**Table 5:** Permanence in percentage of the most relevant non-covalent interactions, with asterisks (*) indicating hydrophobic interactions between the atoms of compound **8b** and the amino acids or nucleotides of the 1T8I receptor.

	Arg364	Lys532	DA113	DC111	DC112	DG12	DT10
O	18%	55%					17%
O1			6%	5%	91%		
O2	92%					9%	
C7							9% *
N2					97%		

**Table 6:** Overview of best poses produced applying molecular docking simulation and then used as starting structures for the four molecular dynamics simulations. The data repository links are also added.

Label	Name of Data File	File Type	Identifier in Data Repository (Figshare)
Data file 1	htop1p-8a	Best pose (.pdb)	https://figshare.com/ndownloader/files/42792178 (accessed on 18 October 2023)
Data file 2	htop1p-8b	Best pose (.pdb)	https://figshare.com/ndownloader/files/42792181 (accessed on 18 October 2023)
Data file 3	htop1p-4a	Best pose (.pdb)	https://figshare.com/ndownloader/files/42792172 (accessed on 18 October 2023)
Data file 4	htop1p-4b	Best pose (.pdb)	https://figshare.com/ndownloader/files/42792175 (accessed on 18 October 2023)

## Data Availability

Data supporting the findings of this study are available within the article. In particular, Table 6 shows the starting structures (best poses obtained from molecular docking simulations) of the molecular dynamics simulations.
